# Yoga for Multiple Sclerosis: A Systematic Review and Meta-Analysis

**DOI:** 10.1371/journal.pone.0112414

**Published:** 2014-11-12

**Authors:** Holger Cramer, Romy Lauche, Hoda Azizi, Gustav Dobos, Jost Langhorst

**Affiliations:** 1 Department of Internal and Integrative Medicine, Kliniken Essen-Mitte, Faculty of Medicine, University of Duisburg-Essen, Essen, Germany; 2 Department of Complementary Medicine, Mashhad University of Medical Sciences, Mashhad, Iran; University of Chieti, Italy

## Abstract

While yoga seems to be effective in a number of neuropsychiatric disorders, the evidence of efficacy in multiple sclerosis remains unclear. The aim of this review was to systematically assess and meta-analyze the available data on efficacy and safety of yoga in patients with multiple sclerosis. Medline/PubMed, Scopus, the Cochrane Central Register of Controlled Trials, PsycINFO, CAM-Quest, CAMbase, and IndMED were searched through March 2014. Randomized controlled trials (RCTs) of yoga for patients with multiple sclerosis were included if they assessed health-related quality of life, fatigue, and/or mobility. Mood, cognitive function, and safety were defined as secondary outcome measures. Risk of bias was assessed using the Cochrane tool. Seven RCTs with a total of 670 patients were included. Evidence for short-term effects of yoga compared to usual care were found for fatigue (standardized mean difference [SMD] = −0.52; 95% confidence intervals (CI) = −1.02 to −0.02; p = 0.04; heterogeneity: I^2^ = 60%; Chi^2^ = 7.43; p = 0.06) and mood (SMD = −0.55; 95%CI = −0.96 to −0.13; p = 0.01; heterogeneity: I^2^ = 0%; Chi^2^ = 1.25; p = 0.53), but not for health-related quality of life, muscle function, or cognitive function. The effects on fatigue and mood were not robust against bias. No short-term or longer term effects of yoga compared to exercise were found. Yoga was not associated with serious adverse events. In conclusion, since no methodological sound evidence was found, no recommendation can be made regarding yoga as a routine intervention for patients with multiple sclerosis. Yoga might be considered a treatment option for patients who are not adherent to recommended exercise regimens.

## Introduction

Multiple sclerosis is the most common chronic autoimmune inflammatory disease of the central nervous system and the leading cause of disability in young adults [1], [2]. Multiple sclerosis is mainly characterized by impaired health-related quality of life, fatigue, and reduced mobility [1]–[3]. Other common symptoms include cognitive impairment, depression, and emotional lability [1], [2].

Yoga is rooted in Indian philosophy and has been a part of traditional Indian spiritual practice for millennia [4]. Yoga traditionally is a complex intervention that comprises not only physical activity but also advice for ethical lifestyle, spiritual practice, breathing exercises, and meditation. While the ultimate goal of traditional yoga has been described as uniting mind, body, and spirit, yoga has become a popular means to promote physical and mental well-being [4], [5]. In North America and Europe, yoga is most often associated with physical postures (asanas), breathing techniques (pranayama), and meditation (dhyana); and different yoga forms have emerged that put varying focus on physical and mental practices [4]. In North America and Europe, yoga is gaining increased popularity as a therapeutic method. About 14 million adult Americans (more than 6% of the population) reported that yoga had been recommended to them by a physician or therapist [6]. Indeed, about half of American yoga practitioners (more than 13 million people) reported that they had started practice explicitly to improve their health [7], [8].

While systematic reviews and meta-analyses have evaluated the efficacy and safety of yoga for a number neuropsychiatric disorders [9]–[11], the evidence of efficacy of yoga in multiple sclerosis has not yet been systematically assessed. Thus, the aim of this review was to systematically evaluate and meta-analyze the available data on efficacy and safety of yoga in improving health-related quality of life, fatigue, mobility, mood, and cognitive function in patients with multiple sclerosis.

## Methods

This review was planned and conducted in accordance with the Preferred Reporting Items for Systematic Reviews and Meta-Analyses (PRISMA) guidelines [12] (Checklist S1) and recommendations of the Cochrane Collaboration [13]. The review protocol was developed a priori and not modified during the conduct of the review.

### Eligibility criteria

#### Types of studies

Randomized controlled trials (RCTs), randomized cross-over studies, and cluster-randomized trials were eligible. No language restrictions were applied.

#### Types of participants

Studies on adult patients (≥18 years) with a diagnosis of multiple sclerosis were eligible.

#### Types of interventions

*Experimental:* Studies on yoga interventions including at least one of the following: physical activity, breath control, meditation, and/or lifestyle advice (based on yoga theory and/or traditional yoga practices) were eligible. No restrictions were made regarding yoga tradition, length, frequency or duration of the program. Studies on multimodal interventions that include yoga amongst others were excluded. Studies allowing individual co-interventions were eligible.

*Control:* Studies comparing yoga to usual care, exercise, or other active non-pharmacological control interventions were eligible.

#### Types of outcome measures

To be eligible for inclusion, studies had to assess at least one primary outcome as recommended by an international, multi-disciplinary consensus meeting [14]:

1. Health-related quality of life, assessed using validated disease-specific instruments such as the Multiple Sclerosis Impact Scale or the Multiple Sclerosis Quality of Life Scale; or generic instruments. Where available, disease-specific instruments were preferred.
2. Fatigue, assessed using validated instruments such as the Fatigue Severity Scale or the Modified Fatigue Impact Scale.
3. Mobility/muscle function, assessed using objective physician-rated tests such as the 6 minute walk test.

Secondary outcomes included:

1. Mood, assessed using validated instruments for depression or anxiety.
2. Cognitive function, assessed using validated neuropsychological tests.
3. Safety of the intervention, assessed as number of patients with adverse events (AEs).

### Search methods

The search strategy comprised seven electronic databases from their inception through March 01, 2014: Medline/PubMed, Scopus, the Cochrane Central Register of Controlled Trials, PsycINFO, CAM-Quest, CAMbase, and IndMED. The literature search was constructed around search terms for 1. “yoga” and 2. “multiple sclerosis” and adapted for each database as necessary. The complete search strategy for Medline was as follows: (“Multiple Sclerosis”[Mesh] OR “Multiple Sclerosis”[Title/Abstract] OR “MS”[Title/Abstract] OR “Disseminated Sclerosis”[Title/Abstract]) AND (“Yoga”[Mesh] OR “Yoga”[Title/Abstract] OR “Yogic”[Title/Abstract] OR “Asana”[Title/Abstract] OR “Pranayama”[Title/Abstract] OR “Dhyana”[Title/Abstract]). Additionally, reference lists of identified original articles or reviews; and the tables of contents of the International Journal of Yoga Therapy and the Journal of Yoga & Physical Therapy were searched manually.

Two reviewers independently screening and selected abstracts; potentially eligible articles were read in full by two reviewers. Disagreements were settled through a discussion with a third reviewer until consensus was reached. If necessary, additional information was obtained from the authors of the primary study.

### Data extraction and management

Two reviewers independently extracted data on patients (e.g. age, gender, ethnicity), methods (e.g. randomization, allocation concealment), interventions (e.g. yoga type, frequency, and duration), control interventions (e.g. type, frequency, duration), outcomes (e.g. outcome measures, assessment time points), and results using an a priori developed data extraction form. Discrepancies were discussed with a third reviewer until consensus was reached.

### Assessment of risk of bias in individual studies

Two reviewers independently assessed risk of bias using the Cochrane risk of bias tool [13]. This tool assesses risk of bias on seven domains: random sequence generation, allocation concealment, blinding of participants and personnel, blinding of outcome assessment, incomplete outcome data, selective reporting, and other sources of bias. For each domain, risk of bias was assessed as low; unclear; or high risk of bias. Discrepancies were discussed with a third reviewer until consensus was achieved.

### Data analysis

#### Assessment of overall effect size

Separate meta-analyses were conducted for comparisons of yoga to different control interventions. Meta-analyses were conducted using Review Manager 5 software (Version 5.1, The Nordic Cochrane Centre, Copenhagen) by random effects models if at least two studies assessing this specific outcome were available. For continuous outcomes, standardized mean differences (SMD) with 95% confidence intervals (CI) were calculated as the difference in means between groups divided by the pooled standard deviation using Hedges’s correction for small study samples [13]. Where no standard deviations were available, they were calculated from standard errors, confidence intervals or t-values [13], or attempts were made to obtain the missing data from the trial authors by email.

A positive SMD (i.e. higher values in the yoga group) was defined to indicate beneficial effects of yoga compared to the control intervention for quality of life, mobility, and cognitive function while a negative SMD (i.e. lower values in the yoga group) was defined to indicate beneficial effects for fatigue and mood. If necessary, values were inverted [13].

Cohen’s categories were used to evaluate the magnitude of the overall effect size with SMD = 0.2–0.5: small; SMD = 0.5–0.8: medium; and SMD>0.8: large effect sizes [15].

### Assessment of heterogeneity

Statistical heterogeneity between studies was analyzed using the I^2^ statistics, a measure of how much variance between studies can be attributed to differences between studies rather than chance. The magnitude of heterogeneity was categorized as (1) I^2^ = 0–24%: low heterogeneity; I^2^ = 25–49%: moderate; I^2^ = 50–74%: substantial; and I^2^ = 75–100%: considerable [13], [16]. The Chi^2^ test was used to assess whether differences in results are compatible with chance alone. Given the low power of this test when only few studies or studies with low sample size are included in a meta-analysis, a P-value≤0.10 was regarded to indicate significant heterogeneity [13].

### Subgroup and sensitivity analyses

Subgroup analyses were planned for the type of yoga intervention (yoga interventions including physical postures vs. yoga interventions without physical postures). As all included studies comprised yoga postures, subgroup analyses could not be performed.

To test the robustness of significant results, sensitivity analyses were conducted for studies with high versus low risk of bias at the following domains: selection bias (random sequence generation and allocation concealment), detection bias (blinding of outcome assessment), and attrition bias (incomplete outcome data).

If present in the respective meta-analysis, subgroup and sensitivity analyses were also used to explore possible reasons for statistical heterogeneity.

### Risk of bias across studies

If at least 10 studies were included in a meta-analysis, assessment of publication bias was originally planned by using funnel plots generated using Review Manager software [13], [17]. As less than 10 studies were included in each meta-analysis, funnel plots could not be analyzed.

## Results

### Literature search

**Figure 1:**
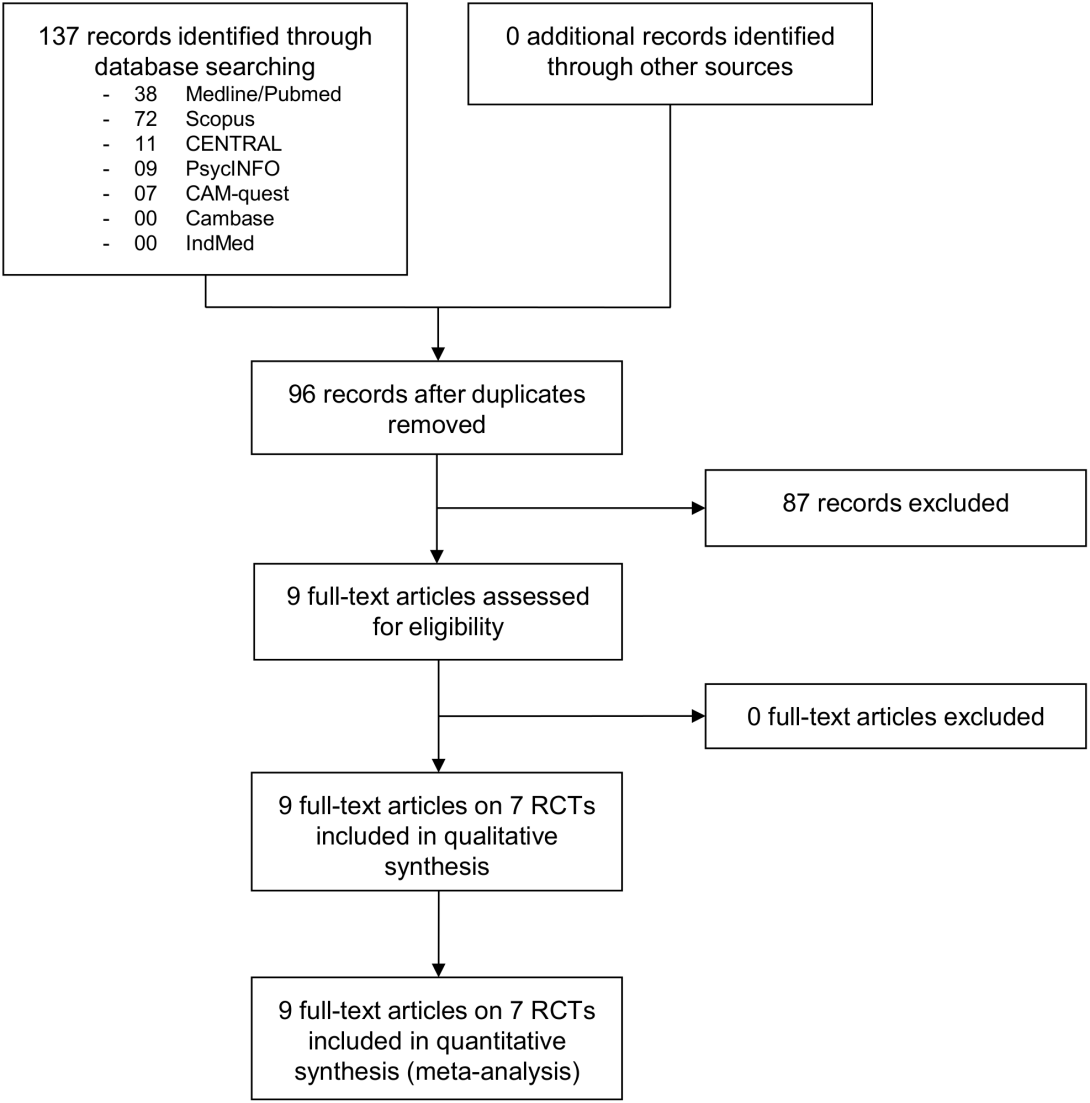
Flowchart of the results of the literature search.

The literature search retrieved 96 non-duplicate records of which 87 were excluded on the basis of title and abstract because they did not meet all pre-defined inclusion criteria (Table S1). Nine full-text articles were assessed for eligibility [18]–[26]. All nine full-text articles reporting on seven RCTs involving a total of 670 patients met the inclusion criteria and were included in the qualitative analysis and meta-analysis (Figure 1). One full-text article was published in Persian [25], the others in English.

### Study characteristics

Characteristics of the sample, interventions, outcome assessment, and results are shown in Table 1.

**Table 1:** Characteristics of the included studies.

Reference	Origin	Sample	Intervention	Control group	Outcomeassessmenttime point	Outcomemeasures:	Resultspost-intervention (between groupdifferences, if nototherwise stated)
	Country; Recruitedfrom	Sample size; diagnosis; current treatment; mean age; ethnicity	Intervention; programlength; frequency; duration	Intervention; programlength; frequency; duration	Post-intervention; longestfollow-up	1. Quality of life; 2. Fatigue; 3. Muscle function/mobility (walking distance); 4. Mood; 5. Cognitive function; 6. Safety	1. Quality of life; 2. Fatigue; 3. Musclefunction/mobility (walking distance); 4. Mood; 5. Cognitive function; 6. Safety
Ahmadi,2010, 2013 [18], [19]	Iran;physiotherapyclinic	31 women; MS diagnosed by physician; diseasemodifying drugs were allowed; 35.2±9.0 years; ethnicity NR	Hatha Yoga; 8 weeks; 3x/weeks; 60–70 min each (postures, breathing techniques, meditation)	Control group 1) Exercise (treadmill training) 8 weeks; 3x/weeks; 30 min each; Control group 2) Usual care	8 weeks	1. MSQOL54: physical healthcomposite, mental health composite; 2. FSS; 3. 10-mtimed walk test, 2-min walk test; 4. BDI, BAI; 5. NA; 6. Exacerbation	1. Sign. improvements in Yoga, but not in Usual care; 2. Sign. improvements in Yoga and Exercise, but not in Usual care; 3. Sign. improvements in Yoga and Exercise, but not in Usual care; 4. BDI,BAI: sign. Improvements in Yogaand Exercise, but not in Usual care; 5. NA; 6. No exacerbations
Doulatabad 2013 [20]	Iran; university hospital	60 women; MS not in acute phase; current treatment NR; 31.6±8.0 years; ethnicity NR	Hatha Yoga; 12 weeks; 2x/week; 60–90 min. each (postures, breathing techniques, meditation)	Usual care	12 weeks	1. MSQOL54;2.-6. NA	1. Sign. improvements in Yoga, but not in Usual care; 2.-6. NA
Garret 2013[21], [22]	Ireland; Multiple Sclerosis Society of Ireland	314; MS diagnosed by physician or neurologist;no steroid treatment; 48.8±11.0 years; ethnicity NR	Yoga, not predefined; 10 weeks; 1x/week; 1 hour each (postures, breathing techniques, relaxation)	Control group 1)Physiotherapist led exercise(aerobic, resistance); 10 weeks; 1x/week; 1 hour each;Control group 2) Fitness instructor led exercise(aerobic, resistance); 10 weeks; 1x/week; 1 hour each; Control group 3) Usual care	12 weeks;24 weeks	1. MSIS-29v2: physical health composite, mental health composite; 2. MFIS; 3. 6-minwalk test; 4.-5. NA; 6. Drop outs due to medicalreasons	1. Sign. improvements in Yoga and Exercise groups, but not in Usual care; sign. larger improvement in Yogacompared to Usual care for mental health composite only; 2. Sign. improvements in Yoga and Exercise groups, but not in Usual care; sign. larger improvement in Yogacompared to Usual care; 3. NS; 4.-5. NA; 6. N = 3/77 in Yoga, N = 4/80 in Physiotherapist led exercise; N = 5/86 in Fitness instructor led exercise, N = 8/71 in Usual care
Hogan 2014 [23]	Ireland; Multiple Sclerosis Society of Ireland	146; MS diagnosed by physician orneurologist; no steroidtreatment; 54.4 years; ethnicity NR	Yoga, not predefined; 10 weeks; 1x/week; 1 hour each (postures, breathing techniques, relaxation)	Control group 1) Individual physiotherapy (aerobic, resistance exercise); 10 weeks;1x/week; 1 hour each; Control group 2) Group led physiotherapy (aerobic, resistance exercise); 10 weeks; 1x/week; 1 hour each;Control group 3) Usual care	12 weeks	1. MSIS-29v2: physical health composite, mental healthcomposite; 2. MFIS; 3. 6-minwalk test; 4.-5. NA; 6. Drop outs due to medical reasons	1. Sign. improvements in Group Physiotherapy, but not Yoga or Usual Care; Sign. improvements in individual physiotherapy for physical health composite only; 2. Sign. improvements in exercise groups, but not Yoga or Usual Care; 3. Sign. improvements in group physiotherapy, but not Yoga or Usual Care; 4.-5. NA; 6. N = 0/16 in Yoga, N = 2/66 in Group Physiotherapy; N = 2/45 in Individual Physiotherapy, N = 1/19 in Usual care
Oken 2004[24]	North America; adverts and newsletters of MS societies	69 patients with MS; medical records; no change in CNS-active medications; 49.0 years; ethnicity NR	Iyengar yoga; 6 months; 1x/week; 90 min each; daily home practice(postures, breathing techniques, relaxation)	Control group 1) Aerobic exercise; 6 months;1x/week; 90 min each; daily home practice;Control group 2) Usual care	6 months	1. SF-36: physical healthcomposite, mental healthcomposite; 2. MFI; 3. 25-foottimed walk test; 4. POMS; CES-D; STAI; 5. Attention (Stroop Color and Word Test, modified Useful Field of View task, adapted attentional shift task, PASAT, Wechsler Memory Scales III Logical Memory, Wechsler Adult IntelligenceScale III Similarities); alertness (EEG, SSS); 5. NA; 6. Adverse events	1.-4. NS; 5. NA; 6. 3 surgeries; 1 exacerbation in Yoga and Exercise each
Rahnama 2011[25]	Iran; Multiple Sclerosis Foundation	30 women with MS; under medication therapy; 33.4 years, ethnicity NR	Yoga; 8 weeks; 2x/week; 60–75 min each; 1x/week home practice (postures, relaxation)	Usual care	8 weeks	1.-3. NA; 4. BDI; 5.-6. NA	1.-3. NA; 4. Sign. improvement in Yoga, but not in Usual care; 5.-6. NA
Velikonja 2010[26]	Slovenia; recruitment NR	20 patients with MS; primary or secondary progressive MS; current treatment NR; age between 26 and 50 years; ethnicity NR	Hatha Yoga; 10 weeks; 1x/week (postures, breathing techniques)	Exercise (Sports climbing)	10 weeks	1. NA; 2. MFIS; 3. NA; 4. CES-D 5. Executivefunctions (NAB, TOL), attention (d2CP); 6. NA	1. NA; 2. Sign. improvement in Exercise, but not in Yoga; 3. NA; 4. NS; 5. Sign. improvement in Yoga, but not in Exercise; 6. NA

Abbreviations: BAI – Beck Anxiety Inventory; BDI – Beck Depression Inventory; CES-D - Center for Epidemiological Studies Depression Scale; d2CP – Brickenkamp d2 test concentration performance; FSS – Fatigue Severity Scale; MAS – Modified Ashworth Scale; MFI – Multidimensional Fatigue Inventory; MFIS – Modified Fatigue Impact Scale; MS – multiple sclerosis; MSIS-29v2– Multiple Sclerosis Impact Scale-29, version2; MSQOL54 - Multiple Sclerosis Quality of Life 54; NA – Not assessed; NAB – Neuropsychological Assessment Battery; NR – not reported; NS – not significant; PASAT – Paced Auditory Serial Addition Test; POMS – Profile of Mood States; SF-36 - Short Form-36 Health Survey; SSS – Stanford Sleepiness Scale; STAI – State Trait Anxiety Inventory; TOL – Tower of London Test.

#### Study and participant characteristics

Of the seven studies that were included, three originated from Iran [18]–[20], [25]; two from Ireland [21]–[23]; and one each from the USA [24]; and Slovenia [26]. Patients were recruited from hospitals [18]–[20], multiple sclerosis societies [21]–[24] or multiple sclerosis foundations [25]. Only three studies reported on the type of multiple sclerosis, all three studies included patients with all types of multiple sclerosis [21]–[23], [26]. The sample size ranged from 20 to 314 with a median of 60. Participant’s mean age ranged from 31.6 to 54.4 years with a median of 42 years. Between 75.2% and 100.0% of participants were female (median 96.5%). Ethnicity was not reported in any study.

#### Intervention characteristics

Three studies used Hatha Yoga [18]–[20], [26]; one used Iyengar Yoga [24]; two did not predefine the yoga intervention but let the yoga teachers decide on content of the intervention [21]–[23]; and one did not report the yoga style used [25]. All seven studies used yoga postures and meditation or relaxation; six also used yogic breathing techniques [18]–[24], [26]. The duration of yoga programs ranged from eight weeks to six months with a median of 10 weeks; frequency of yoga interventions ranged from one to three (median: 1) weekly yoga sessions of 60 to 90 (median: 66.25) minutes length. Six studies compared yoga to usual care or no specific treatment [18]–[25]. Five studies compared yoga to exercise including treadmill training [18], [19]; aerobic exercise [24]; mixed aerobic and resistance exercise [21]–[23]; or sports climbing [26]. Except for one study where the yoga sessions were longer in duration than the exercise session [18], [19], the yoga and exercise interventions were exactly matched for program length, and frequency and duration of the sessions.

#### Outcome measures

All seven studies assessed outcomes immediately after the end of the intervention; one study also assessed longer-term effects 12 weeks after the end of the intervention [21], [22]. Health-related quality of life was assessed in five studies using the Multiple Sclerosis Quality of Life 54 [18]–[20], the Multiple Sclerosis Impact Scale-29 [21]–[23], or the Short Form-36 Health Survey [24]. Five studies assessed fatigue with the Fatigue Severity Scale [18], [19], the Modified Fatigue Impact Scale [21]–[23], [26], or the Multidimensional Fatigue Inventory [24]. Mobility was assessed using the 10-m timed walk test [18], [19], the 25-foot timed walk test [24], the 2-minute walk test [18], [19], or the 6-minute walk test [21]–[23]. Mood was assessed in three studies using the Beck Depression Inventory [18], [19], [25], the Beck Anxiety Inventory [18], [19], the Profile of Mood States [24], the Center for Epidemiological Studies Depression Scale [24], [26], and/or the State Trait Anxiety Inventory [24]. Two studies assessed cognitive function using standardized neuropsychological test batteries [24], [26] (table 2). Four studies reported safety-related data [18], [19], [21], [22], [23], [24].

**Table 2:** Sensitivity analysis: effect sizes when only trials with low risk of detection bias were included.

Comparison/Outcome	No. of studies	No. ofpatients (yoga)	No. ofpatients(control)	Standardizedmean difference(95% confidence interval)	P(overall effect)	HeterogeneityI^2^; Chi^2^; P
**Yoga versus usual care**						
Quality of life	3	98	84	−0.00 (−0.30; 0.29)	0.98	0%; 0.17; 0.92
Fatigue	3	98	84	−0.32 (−0.72; 0.08)	0.12	36%; 3.14; 0.21
Mobility	1	22	20	−0.20 (−0.80; 0.41)	0.52	-
Mood	1	22	20	−0.63 (−1.25; −0.01)	0.05	-
**Yoga versus exercise**						
Quality of life	3	98	230	0.09 (−0.15; 0.34)	0.46	0%; 0.16; 0.92
Fatigue	3	98	230	0.05(−0.21; 0.31)	0.70	5%; 2.11; 0.35
Mobility	1	22	16	−0.20 (−0.85; 0.44)	0.54	-
Mood	1	22	16	0.31 (−0.34; 0.96)	0.35	-
Cognitive function	1	22	16	0.26 (−0.39; 0.91)	0.43	-

### Risk of bias in individual studies

**Figure 2:**
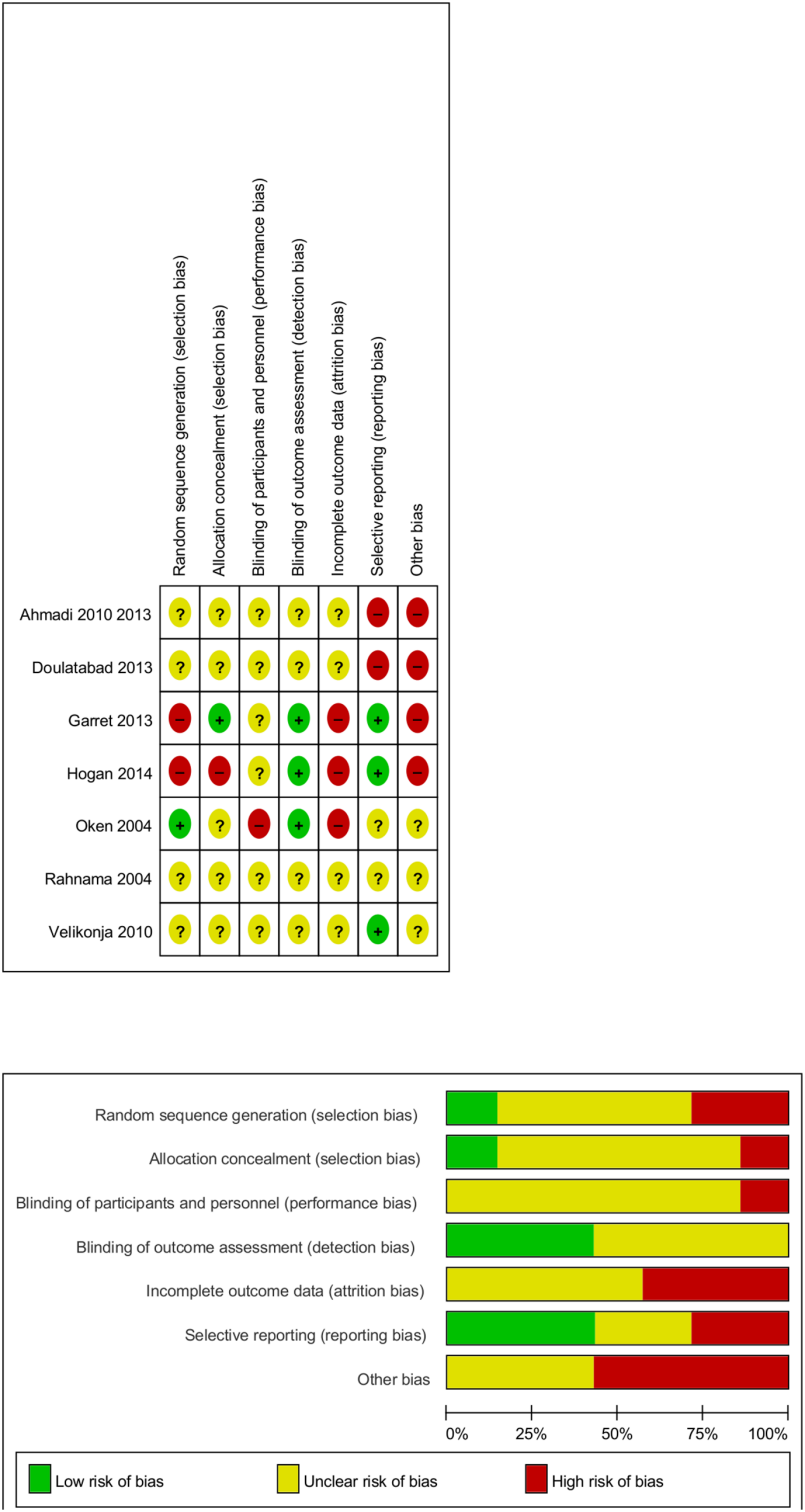
Risk of bias for each criterion for each included study (top) and risk of bias for each criterion presented as percentages across all included studies (bottom).

Risk of bias in individual studies is shown in figure 2. One study each had reported adequate random sequence generation [24] or allocation concealment [21], [22]; and three studies each reported adequate blinding of outcome assessement [21]–[24] or were free of suspected selective reported [21], [22], [26].

### Analysis of overall effect

#### Primary outcomes

**Figure 3:**
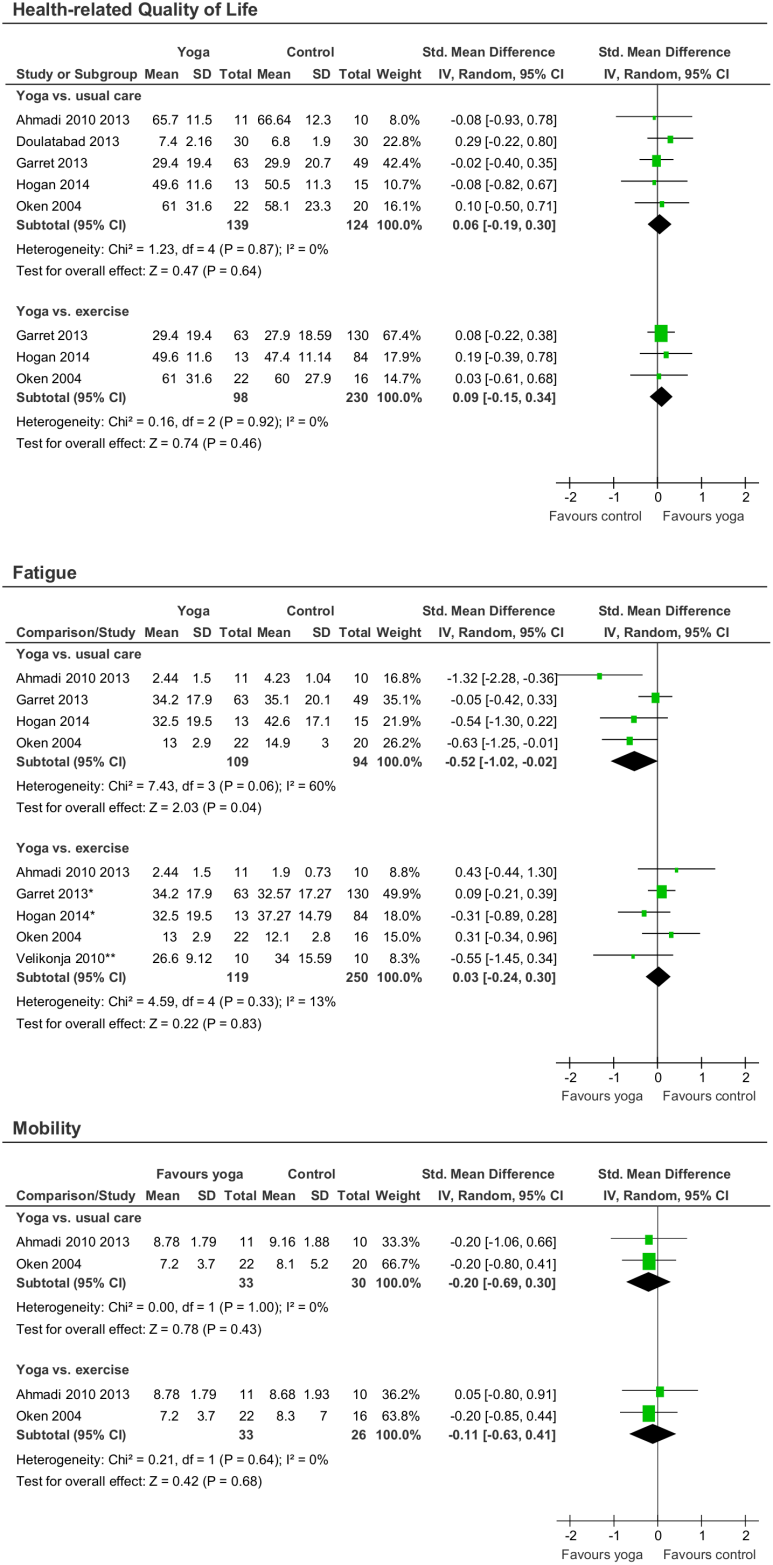
Effect sizes for the primary outcomes quality of life, fatigue, and mobility. *both exercise groups combined; **means and standard deviations provided upon request.

Meta-analyses revealed evidence for short-term effects of yoga compared to usual care on fatigue (SMD = −0.52; 95% CI = −1.02 to −0.02; p = 0.04) but not on health-related quality of life (SMD = 0.06; 95% CI = −0.19 to 0.30; p = 0.64) or mobility (SMD = −0.20; 95% CI = −0.69 to 0.30; p = 0.42). No evidence for effects of yoga compared to exercise was found for fatigue (SMD = 0.03; 95% CI = −0.24 to 0.30; p = 0.83), health-related quality of life (SMD = 0.09; 95% CI = −0.15 to 0.34; p = 0.46), or mobility (SMD = −0.11; 95% CI = −0.63 to 0.41; p = 0.68) was found (figure 3). while no meta-analysis was possible, based on single RCTs no evidence for longer-term effects of yoga compared to exercise on health-related quality of life, fatigue, or mobility was found [21], [22].

#### Secondary outcomes

**Figure 4:**
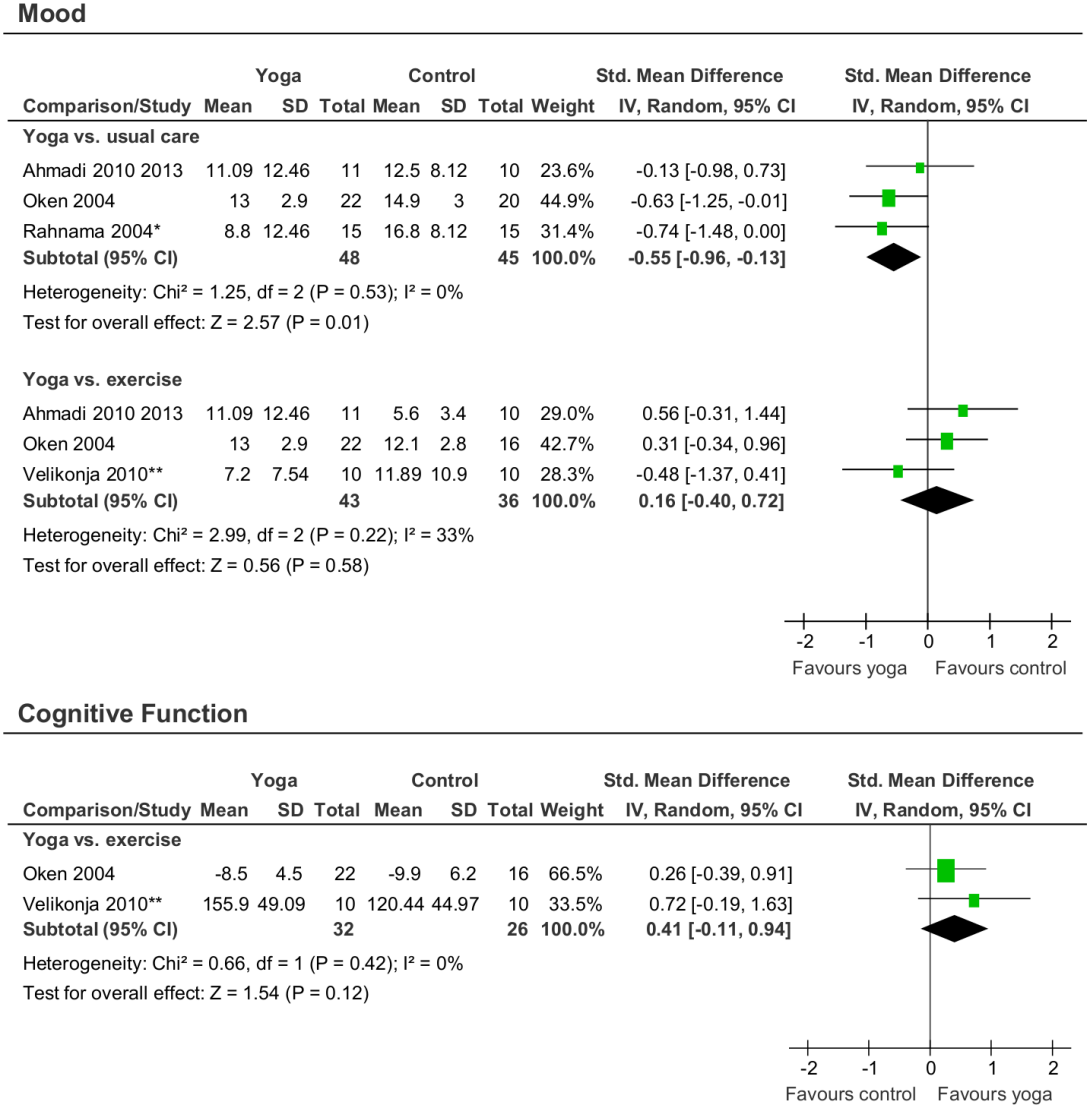
Effect sizes for the secondary outcomes mood and cognitive function. *standard deviation imputed from other studies; **means and standard deviations provided upon request.

Evidence for short-term effects of yoga compared to usual care was found for mood (SMD = −0.55; 95% CI−0.96, −0.12; p = 0.01) (figure 4). No evidence for short-term effects of yoga compared to exercise on mood (SMD = 0.16; 95% CI−0.40 to 0.72; p = 0.58) or cognitive function (SMD = 0.41; 95% CI−0.11 to 0.94; p = 0.12) was found (figure 4, table 2).

#### Safety

Four studies reported safety-related data. One of those reported adverse events: one exacerbation in each group occurred, and four further adverse events were reported that were not related to the study interventions [24]. In another study, no exacerbation occurred in any of the three groups [18], [19]. In another study, three patients in the yoga group were lost to follow-up after the intervention due to medical reasons (including exacerbations) compared to four, five, and eight patients in the physiotherapist-led exercise, fitness instructor-led exercise, and usual care group, respectively [21], [22]. In the fourth study, zero, two, three, and one patients were lost to follow-up after the intervention due to medical reasons in the yoga, group physiotherapy, individual physiotherapy, and control group, respectively [23].

### Sensitivity analyses

Since no study had low risk of selection or attrition bias, no effect remained significant in sensitivity analyses for low risk of selection or attrition bias. In studies with low risk of detection bias, the effect of yoga compared to usual care on mood did not change substantially (SMD = −0.63; 95% CI−1.25, −0.01; p = 0.05). No other effects remained significant (table 2).

## Discussion

### Summary of evidence

In this systematic review of seven randomized trials on yoga for multiple sclerosis, evidence for positive short-term effects of yoga on fatigue and mood, but not on more objective physician-rated outcomes such as mobility or cognitive function were found. No effect was robust against potential methodological bias. Although the overall high risk of bias hinders definite conclusions, yoga seems to be equally effective as exercise interventions in improving both patient-reported and physician-rated outcomes.

Safety of the intervention was insufficiently reported. Specifically, only one study explicitly assessed adverse events. However, exacerbations of multiple sclerosis were fewer or equal in number in the groups compared to the usual care or exercise groups. This is in line with previous cross-sectional studies [27], [28] and systematic reviews of yoga interventions in other patient populations that found no evidence for serious yoga-associated adverse events [29]–[33]. It should however be considered that yoga has occasionally been associated with serious adverse events in case studies [34].

### Agreements with prior systematic reviews

No systematic review specifically on yoga for multiple sclerosis was available. However, a recent review on studies on mind-body medicine for multiple sclerosis published until March 2012 [35] included one RCT on yoga [24]. This review concluded that while yoga improved multiple sclerosis-related fatigue with fewer side effects than conventional treatment, more research was needed to draw definite conclusions. A recent systematic review on exercise for multiple sclerosis concluded that exercise therapy including yoga [24] may have beneficial effects on patients with multiple sclerosis without adverse events and might thus be recommended for the rehabilitation of multiple sclerosis patients [36]. A systematic review on yoga for fatigue that also conducted a meta-analysis included two trials on multiple sclerosis [24], [26] amongst trials other conditions. This meta-analysis found moderate evidence for effects of yoga on fatigue with larger effect sizes in non-cancer populations [37]. The recent guideline on complementary and alternative medicine in multiple sclerosis by the American Academy of Neurology included a systematic review on studies of yoga for multiple sclerosis [38], [39]. This review included four studies [19], [21], [24], [26] and concluded that, mainly due the low power of the available trials, the data were inadequate to assess the effect of yoga on disability, spasticity, fatigue, cognition, mood, balance, or walking speed in patients with multiple sclerosis [38], [39].

### External and internal validity

Mainly patients from the Middle East and Europe were included. Given that ethnical and geographical factors influence the incidence and severity of multiple sclerosis [1], [2], the applicability of these findings in other geographical regions is limited. Moreover, since mainly female patients were included, the results might not be fully applicable to male patients. Four studies did not report on the eligible types of multiple sclerosis [18]–[20], [24], [25]. This further limits the applicability of the results.

Overall, risk of bias of the included studies was unclear or high. Most importantly, only one study each reported adequate random sequence generation [24] or allocation concealment [21], [22]. As inadequate allocation concealment has been empirically demonstrated to be the most important source of bias in RCTs [40], this strongly limits the interpretability of results. In sensitivity analyses, no effect was robust against all potential sources of bias. The internal validity of the results is thus limited.

### Strengths and weaknesses

To the best of our knowledge, this is the first systematic review and/or meta-analysis available on yoga for patients with multiple sclerosis. Strengths of this review include the comprehensive literature search without language restrictions and the assessment of applicability of the results [41]. The primary limitation of this review is the paucity of eligible studies, rendering subgroup analyses impossible and resulting in a relatively limited overall sample size. Another major limitation is the insufficient reporting and/or low methodological quality of the included studies, limiting the interpretability of the results. Moreover, the reliability of the Cochrane risk of bias tool in systematic reviews on clinical trials of behavioral interventions has been questioned. It has been demonstrated that there is poor inter-rater reliability for several of the domains, specifically for the domain ‘blinding of participants and personnel’ [42]. Since it is generally regarded impossible to blind patients and therapists in trials of behavioral interventions such as yoga, different research groups tend to rate this domains differently if no information on blinding is provided. Accordingly, this domain was not used in sensitivity analyses. The applicability of the findings was limited. As only one study reported longer-term effects [21], [22], the results of this review are only applicable to the short-term. No unpublished studies or studies published in ‘grey literature’ were included. The usefulness of including unpublished trials is still under debate [13]. Meant to address publication bias, only few unpublished trials can normally be located for systematic reviews and the located studies may be an unrepresentative sample of all unpublished studies [13], [43]. Investigators are often unwilling to provide unfavorable results; thus publication bias may still remain an issue [13]. Moreover, unpublished studies tend to be of lower methodological quality than published studies [44] and normally lack peer-review [13].

### Implications for further research

Given that the main drawback of this review was the insufficient reporting of trial methodology, authors of prospect research should improve the reporting of yoga trials and follow commonly accepted reporting guidelines (e.g. CONSORT) [45]. Future studies should ensure rigorous methodology, mainly adequate randomization, allocation concealment, intention-to-treat analysis, and blinding of at least outcome assessors [45].

### Implications for clinical practice

While this review found evidence for effects of yoga on fatigue and mood in patients with multiple sclerosis, due to the insufficient reporting of the included trials and the limited external validity, this evidence should be applied in clinical practice with care. Thus, no recommendation can be made regarding yoga as a routine intervention for patients with multiple sclerosis at this point. Given that yoga was not associated with severe adverse events, its practice needs not be discouraged in this patient population. Given its evident effectiveness [36], exercise is strongly encouraged by international guideline for the treatment of multiple sclerosis-associated symptoms, especially for improving fatigue [46], [47], [48]. Since fatigue seems to be equally improved by yoga and exercise, yoga might be considered a treatment option for patients who are not adherent to recommended exercise regimens.

## Supporting Information

Table S1 Records excluded after screening of title and abstract with reasons for exclusion.
(PDF)

Checklist S1 **PRISMA Checklist.**
(DOC)

## References

[1] Compston A, Coles A (2008) Multiple sclerosis. Lancet 372: 1502–1517. 18970977 10.1016/S0140-6736(08)61620-7

[2] Noseworthy JH, Lucchinetti C, Rodriguez M, Weinshenker BG (2000) Multiple sclerosis. N Engl J Med 343: 938–952. 11006371 10.1056/NEJM200009283431307

[3] Riazi A, Hobart JC, Lamping DL, Fitzpatrick R, Freeman JA, et al (2003) Using the SF-36 measure to compare the health impact of multiple sclerosis and Parkinson’s disease with normal population health profiles. J Neurol Neurosurg Psychiatry 74: 710–714. 12754336 10.1136/jnnp.74.6.710 PMC1738466

[4] Feuerstein G (1998) The yoga tradition. Prescott: Hohm Press.

[5] Iyengar BKS (1966) Light on yoga. New York: Schocken Books.

[6] Macy D (2008) Yoga journal releases 2008 “Yoga in America” market study. Yoga Journal. Available: http://www.yogajournal.com/advertise/press_releases/10. Accessed 7 March 2014.

[7] Barnes PM, Bloom B, Nahin RL (2008) Complementary and alternative medicine use among adults and children: United States, 2007. Natl Health Stat Report (12): 1–23. 19361005

[8] Barnes PM, Powell-Griner E, McFann K, Nahin RL (2004) Complementary and alternative medicine use among adults: United States, 2002. Advance data (343): 1–19. 15188733

[9] Vancampfort D, Vansteelandt K, Scheewe T, Probst M, Knapen J, et al (2012) Yoga in schizophrenia: a systematic review of randomised controlled trials. Acta Psychiatr Scand 126: 12–20. 22486714 10.1111/j.1600-0447.2012.01865.x

[10] Cramer H, Lauche R, Klose P, Langhorst J, Dobos G (2013) Yoga for schizophrenia: a systematic review and meta-analysis. BMC Psychiatry 13: 32. 23327116 10.1186/1471-244X-13-32 PMC3608162

[11] Cramer H, Lauche R, Langhorst J, Dobos G (2013) Yoga for depression: a systematic review and meta-analysis. Depress Anxiety 20: 1068–83. 10.1002/da.22166 23922209

[12] Moher D, Liberati A, Tetzlaff J, Altman DG (2009) Preferred reporting items for systematic reviews and meta-analyses: the PRISMA statement. BMJ 339: b2535. 19622551 10.1136/bmj.b2535 PMC2714657

[13] Higgins JPT, Green S (2008) Cochrane handbook for systematic reviews of interventions. West Sussex: John Wiley & Sons Ltd.

[14] Paul L, Crosbie J, Coote S, Dixon D, Hale L, et al. (2013) Outcome measures for studies of exercise in multiple sclerosis: recommendations from an international, multi-disciplinary consensus meeting. 29th Congress on the European Committee for Treatment and Research in Multiple Sclerosis. Available: http://registrationakmch/einsichtphp?XNABSTRACT_ID=179215&XNSPRACHE_ID=2&XNKONGRESS_ID=195&XNMASKEN_ID=900. Accessed 7 March, 2014.

[15] Cohen J (1988) Statistical Power Analysis for the Behavioral Sciences. Hillsdale: Lawrence Erlbaum Associates.

[16] Higgins JP, Thompson SG, Deeks JJ, Altman DG (2003) Measuring inconsistency in meta-analyses. BMJ 327: 557–560. 12958120 10.1136/bmj.327.7414.557 PMC192859

[17] Egger M, Davey Smith G, Schneider M, Minder C (1997) Bias in meta-analysis detected by a simple, graphical test. BMJ 315: 629–634. 9310563 10.1136/bmj.315.7109.629 PMC2127453

[18] Ahmadi A, Arastoo AA, Nikbakht M, Zahednejad S, Rajabpour M (2013) Comparison of the Effect of 8 weeks Aerobic and Yoga Training on Ambulatory Function, Fatigue and Mood Status in MS Patients. Iran Red Crescent Med J 15: 449–454. 24349740 10.5812/ircmj.3597 PMC3840829

[19] Ahmadi A, Nikbakh M, Arastoo A, Habibi AH (2010) The Effects of a yoga intervention on balance, speed and endurance of walking, fatigue and quality of life in people with multiple sclerosis. J Hum Kinet 23: 71–78.

[20] Doulatabad SN, Nooreyan K, Doulatabad AN, Noubandegani ZM (2012) The effects of pranayama, hatha and raja yoga on physical pain and the quality of life of women with multiple sclerosis. Afr J Tradit Complement Altern Med 10: 49–52. 24082325 PMC3746357

[21] Garrett M, Hogan N, Larkin A, Saunders J, Jakeman P, et al (2013) Exercise in the community for people with minimal gait impairment due to MS: an assessor-blind randomized controlled trial. Mult Scler 19: 782–789. 23128667 10.1177/1352458512461966

[22] Garrett M, Hogan N, Larkin A, Saunders J, Jakeman P, et al (2013) Exercise in the community for people with multiple sclerosis - A follow-up of people with minimal gait impairment. Mult Scler 19: 790–798. 23132904 10.1177/1352458512461390

[23] Hogan N, Kehoe M, Larkin A, Coote S (2014) The Effect of Community Exercise Interventions for People with MS Who Use Bilateral Support for Gait. Mult Scler Int 2014: 109142. 24575302 10.1155/2014/109142 PMC3910069

[24] Oken BS, Kishiyama S, Zajdel D, Bourdette D, Carlsen J, et al (2004) Randomized controlled trial of yoga and exercise in multiple sclerosis. Neurology 62: 2058–2064. 15184614 10.1212/01.wnl.0000129534.88602.5c

[25] Rahnama N, Namazizadeh M, Etemadifar M, Bambaeichi E, Arbabzadeh S, et al (2011) Effects of yoga on depression in women with multiple sclerosis. Journal of Isfahan Medical School 29: 483–490.

[26] Velikonja O, Curic K, Ozura A, Jazbec SS (2010) Influence of sports climbing and yoga on spasticity, cognitive function, mood and fatigue in patients with multiple sclerosis. Clin Neurol Neurosurg 112: 597–601. 20371148 10.1016/j.clineuro.2010.03.006

[27] Cramer H, Lauche R, Langhorst J, Dobos G, Paul A (2013) Quality of life and mental health in patients with chronic diseases who regularly practice yoga and those who do not: a case-control study. Evid Based Complement Alternat Med 2013: 702914. 23840263 10.1155/2013/702914 PMC3690235

[28] Cramer H, Lauche R, Langhorst J, Paul A, Michalsen A, et al (2013) Predictors of yoga use among internal medicine patients BMC Complement Altern Med. 13: 172. 10.1186/1472-6882-13-172 PMC3717114 23849549

[29] Cramer H, Lange S, Klose P, Paul A, Dobos G (2012) Yoga for breast cancer patients and survivors: a systematic review and meta-analysis. BMC cancer 12: 412. 22988934 10.1186/1471-2407-12-412 PMC3527138

[30] Cramer H, Lauche R, Haller H, Dobos G (2013) A systematic review and meta-analysis of yoga for low back pain. Clin J Pain 29: 450–460. 23246998 10.1097/AJP.0b013e31825e1492

[31] Cramer H, Lauche R, Haller H, Dobos G, Michalsen A (2014) A systematic review of yoga for heart disease. Eur J Prev Cardiol. doi:10.1177/2047487314523132. 10.1177/2047487314523132 24491402

[32] Cramer H, Lauche R, Haller H, Steckhan N, Michalsen A, et al (2014) Effects of yoga on cardiovascular disease risk factors: a systematic review and meta-analysis. Int J Cardiol 173: 170–183. 24636547 10.1016/j.ijcard.2014.02.017

[33] Cramer H, Lauche R, Langhorst J, Dobos G (2012) Effectiveness of yoga for menopausal symptoms: a systematic review and meta-analysis of randomized controlled trials. Evid Based Complement Alternat Med 2012: 863905. 23304220 10.1155/2012/863905 PMC3524799

[34] Cramer H, Krucoff C, Dobos G (2013) Adverse Events Associated with Yoga: A Systematic Review of Published Case Reports and Case Series. PLoS ONE 8: e75515. 24146758 10.1371/journal.pone.0075515 PMC3797727

[35] Senders A, Wahbeh H, Spain R, Shinto L (2012) Mind-body medicine for multiple sclerosis: a systematic review. Autoimmune Dis 2012: 567324. 23227313 10.1155/2012/567324 PMC3512214

[36] Sá MJ (2013) Exercise therapy and multiple sclerosis: a systematic review. J Neurol. doi:10.1007/s00415-013-7183-9. 10.1007/s00415-013-7183-9 24263406

[37] Boehm K, Ostermann T, Milazzo S, Bussing A (2012) Effects of yoga interventions on fatigue: a meta-analysis. Evid Based Complement Alternat Med 2012: 124703. 22991569 10.1155/2012/124703 PMC3443845

[38] Yadav V, Bever C Jr, Bowen J, Bowling A, Weinstock-Guttman B, et al (2014) Summary of evidence-based guideline: complementary and alternative medicine in multiple sclerosis: report of the guideline development subcommittee of the American Academy of Neurology. Neurology 82: 1083–1092. 24663230 10.1212/WNL.0000000000000250 PMC3962995

[39] Yadav V, Bever C Jr, Bowen J, Bowling A, Weinstock-Guttman B, et al. (2014) Summary of evidence-based guideline: complementary and alternative medicine in multiple sclerosis: report of the guideline development subcommittee of the American Academy of Neurology. Available: https://www.aan.com/Guidelines/Home/GetGuidelineContent/643. Accessed 5 June, 2014.

[40] Schulz KF, Chalmers I, Hayes RJ, Altman DG (1995) Empirical evidence of bias. Dimensions of methodological quality associated with estimates of treatment effects in controlled trials. JAMA 273: 408–412. 7823387 10.1001/jama.273.5.408

[41] Gartlehner G (2008) Assessment of adverse effects and applicability–two areas not (yet) covered adequately in Cochrane reports. Z Evid Fortbild Qual Gesundhwes 102: 497–502. 19216199 10.1016/j.zefq.2008.08.028

[42] Armijo-Olivo S, Ospina M, da Costa BR, Egger M, Saltaji H, et al (2014) Poor reliability between Cochrane reviewers and blinded external reviewers when applying the Cochrane risk of bias tool in physical therapy trials. PLOS One 9: e96920. 24824199 10.1371/journal.pone.0096920 PMC4019638

[43] Hetherington J, Dickersin K, Chalmers I, Meinert CL (1989) Retrospective and prospective identification of unpublished controlled trials: lessons from a survey of obstetricians and pediatricians. Pediatrics 84: 374–380. 2748270

[44] Egger M, Jüni P, Bartlett C, Holenstein F, Sterne J (2003) How important are comprehensive literature searches and the assessment of trial quality in systematic reviews? Empirical study. Health Technology Assessment 7: 1. 12583822

[45] Schulz KF, Altman DG, Moher D (2010) CONSORT 2010 statement: updated guidelines for reporting parallel group randomized trials. Ann Intern Med 152: 726–732. 20335313 10.7326/0003-4819-152-11-201006010-00232

[46] National Multiple Sclerosis Society (2008) Management of MS-related fatigue. New York: National Multiple Sclerosis Society.

[47] Latimer-Cheung AE, Martin Ginis KA, Hicks AL, Motl RW, Pilutti LA, et al (2013) Development of evidence-informed physical activity guidelines for adults with multiple sclerosis. Arch Phys Med Rehabil 94: 1829–1836. 23770262 10.1016/j.apmr.2013.05.015

[48] National Collaborating Centre for Chronic Conditions (2004) Multiple Sclerosis: National clinical guideline for diagnosis and management in primary and secondary care. London: Royal College of Physicians (UK). 21290636

