# Supplementary material for: Yoga for Multiple Sclerosis: A Systematic Review and Meta-Analysis
Source: PLoS One. 2014 Nov 12;9(11):e112414. doi: 10.1371/journal.pone.0112414 (PMC4229199; doi:10.1371/journal.pone.0112414)
Supplement: Table S1 — Records excluded after screening of title and abstract with reasons for exclusion. (PDF) [file pone.0112414.s001.pdf]

Table S2: Records excluded after screening of title and abstract with reasons for exclusion.

Record	Reason for exclusion
Afonso, 2012 [1]	Not on multiple sclerosis
Alajbegovic, 2011 [2]	Not on yoga; no clinical trial
Amato & Portaccio, 2012 [3]	No clinical trial
Atri, 2012 [4]	Not on yoga
Aung, 2012 [5]	No clinical trial
Berkman, 1999 [6]	No clinical trial
Bernardi, 2001 [7]	Not on multiple sclerosis
Bhavanani, 2012 [8]	Not on multiple sclerosis
Boehm, 2012 [9]	No clinical trial
Bombardier, 2013 [10]	Not on yoga
Bowling, 2010 [11]	No clinical trial
Bowling, 2011 [12]	No clinical trial
Bowman, 1997 [13]	Not on multiple sclerosis
Branas, 2000 [14]	No clinical trial
Brown, 2009 [15]	No clinical trial
Cassileth, 2010 [16]	Not on multiple sclerosis
Chaitow, 2013 [17]	Not on multiple sclerosis
Susan Coote, 2009 [18]	No clinical trial
Coote, 2013 [19]	Not on yoga
Crayton, 2006 [20]	Not on yoga; no clinical trial
Dabhade, 2012 [21]	Not on multiple sclerosis
Dash, 1999 [22]	Not on multiple sclerosis
Eguíluz, 2013 [23]	Not on yoga
Engebretson, 2002 [24]	No clinical trial

Esmonde, 2008 [25]	No clinical trial
Fawcett, 1994 [26]	No clinical trial
Fowler, 2006 [27]	No clinical trial
Fryze, 2006 [28]	No clinical trial
Galantino, 2003 [29]	No clinical trial
Hankin, 2010 [30]	Not on yoga
Harper, 2009 [31]	Not randomized
Hayes, 2010 [32]	No clinical trial
Hessen, 2006 [33]	No clinical trial
Huntley, 2006 [34]	No clinical trial
Jain, 2004 [35]	Not on yoga
Jensen, 2007 [36]	Not on yoga
Jovanov, 2005 [37]	Not on multiple sclerosis
Kabat-Zinn, 2002 [38]	No clinical trial
Latimer-Cheung, 2013 [39]	No clinical trial
Lee, 2012 [40]	Not on multiple sclerosis
Lovera, 2012 [41]	Not on yoga
Mackereth, 2009 [42]	Not on yoga
Madanmohan, 1992 [43]	Not on multiple sclerosis
Maguire, 1996 [44]	Not on yoga
Mailhan, 2012 [45]	No clinical trial
Markil, 2012 [46]	Not on multiple sclerosis
McDonnell, 2011 [47]	No clinical trial
Meyer, 2012 [48]	No clinical trial
Miller, 2006 [49]	No clinical trial

Mishra, 2012 [50]	No clinical trial
Molina-Rueda, 2009 [51]	No clinical trial
Monika, 2012 [52]	Not on multiple sclerosis
Muralikrishnan, 2012 [53]	Not on multiple sclerosis
Nayak, 2003 [54]	No clinical trial
Oken, 2006 [55]	Not on yoga
Ozura, 2011 [56]	No clinical trial
Page, 2003 [57]	No clinical trial
Pal, 2012 [58]	Not on yoga
Patil, 2012 [59]	No clinical trial
Patra, 2010 [60]	Not on multiple sclerosis
Pearce, 2012 [61]	Not on multiple sclerosis
Penner, 2006 [62]	No clinical trial
Piwko, 2007 [63]	No clinical trial
Pozzilli, 2006 [64]	No clinical trial
Pritchard, 2010 [65]	Not randomized
Rae-Grant, 2011 [66]	No clinical trial
Rodgers, 1996 [67]	Not on yoga
Rothenberg, 2003 [68]	No clinical trial
Ryan, 2002 [69]	No clinical trial
Salgado, 2013 [70]	Not randomized
Schwarz, 2008 [71]	No clinical trial
Senders, 2012 [72]	No clinical trial
Shakeel, 2010 [73]	No clinical trial
Shigaki, 2006 [74]	No clinical trial

Shinto, 2006 [75]	No clinical trial
Skovgaard, 2013 [76]	No clinical trial
Stevinson, 2011 [77]	No clinical trial
Tavee, 2012 [78]	Not on yoga
Tavee, 2010 [79]	No clinical trial
Telles, 2013 [80]	Not on multiple sclerosis
Thompson, 2005 [81]	No clinical trial
Vojdani, 2011 [82]	No clinical trial
Wahbeh, 2008 [83]	No clinical trial
Walker, 2007 [84]	No clinical trial
Wiles, 2008 [85]	No clinical trial
Winterholler, 1997 [86]	No clinical trial
Yadav, 2006 [87]	No clinical trial

References:

1. Afonso RF, Hachul H, Kozasa EH, Souza Oliveira D, Goto V, et al. (2012) Yoga decreases insomnia in postmenopausal women: A randomized clinical trial. Menopause (New York, NY). pp. 186-193.
2. Alajbegovic A, Alajbegovic S, Delilovic-Vranic J (2011) Dietary supplements in multiple sclerosis. Acta Clinica Croatica 50: 65-69.
3. Amato MP, Portaccio E (2012) Management options in multiple sclerosis-associated fatigue. Expert Opinion on Pharmacotherapy 13: 207-216.
4. Atri AE, Saeedi M, Sorouri F, Sokhangooy MK (2012) The effect of aquatic exercise program on fatigue in women with multiple sclerosis. Journal of Mazandaran University of Medical Sciences 22: 53-61.
5. Aung SKH, Greenwood MT, Reyes-Campos MJ, Diaz-Toral LG (2012) How do you treat urinary incontinence in your practice? Medical Acupuncture 24: 56-58.
6. Berkman CS, Pignotti MG, Cavallo PF, Holland NJ (1999) Use of alternative treatments by people with multiple sclerosis. Neurorehabilitation and Neural Repair 13: 243-254.
7. Bernardi L, Sleight P, Bandinelli G, Cencetti S, Fattorini L, et al. (2001) Effect of rosary prayer and yoga mantras on autonomic cardiovascular rhythms: comparative study. BMJ 323: 1446-1449.
8. Bhavanani AB, Ramanathan M, Kt H (2012) Immediate effect of mukha bhastrika (a bellows type pranayama) on reaction time in mentally challenged adolescents. Indian J Physiol Pharmacol 56: 174-180.
9. Boehm K, Ostermann T, Milazzo S, Bussing A (2012) Effects of yoga interventions on fatigue: a meta-analysis. Evid Based Complement Alternat Med 2012: 124703.

10. Bombardier CH, Ehde DM, Gibbons LE, Wadhwani R, Sullivan MD, et al. (2013) Telephone-based physical activity counseling for major depression in people with multiple sclerosis. Journal of Consulting and Clinical Psychology 81: 89-99.
11. Bowling AC (2010) Complementary and alternative medicine in multiple sclerosis. CONTINUUM: Lifelong Learning in Neurology 16: 78-89.
12. Bowling AC (2011) Complementary and Alternative Medicine and Multiple Sclerosis. Neurologic Clinics 29: 465-480.
13. Bowman AJ, Clayton RH, Murray A, Reed JW, Subhan MM, et al. (1997) Effects of aerobic exercise training and yoga on the baroreflex in healthy elderly persons. Eur J Clin Invest 27: 443-449.
14. Branas P, Jordan R, Fry-Smith A, Burls A, Hyde C (2000) Treatments for fatigue in multiple sclerosis: a rapid and systematic review. Health Technol Assess 4: 1-61.
15. Brown RP, Gerbarg PL (2009) Yoga breathing, meditation, and longevity. pp. 54-62.
16. Cassileth B (2010) Integrative oncology - Yoga. ONCOLOGY 24.
17. Chaitow L (2013) Recognizing self-regulation. Journal of Bodywork and Movement Therapies 17: 393-394.
18. Coote S, Garrett M, Hogan N, Larkin A, Saunders J (2009) Getting the balance right: A randomised controlled trial of physiotherapy and exercise interventions for ambulatory people with multiple sclerosis. BMC Neurology 9: 34.
19. Coote S, Hogan N, Franklin S (2013) Falls in people with multiple sclerosis who use a walking aid: prevalence, factors, and effect of strength and balance interventions. Arch Phys Med Rehabil 94: 616-621.
20. Crayton HJ, Rossman HS (2006) Managing the symptoms of multiple sclerosis: A multimodal approach. Clinical Therapeutics 28: 445-460.
21. Dabhade AM, Pawar BH, Ghunage MS, Ghunage VM (2012) Effect of pranayama (breathing exercise) on arrhythmias in the human heart. Explore (NY) 8: 12-15.
22. Dash M, Telles S (1999) Yoga training and motor speed based on a finger tapping task. Indian J Physiol Pharmacol 43: 458-462.
23. Eguíluz G, García MB (2013) Use of a time-of-flight camera with an Omek Beckon™ framework to analyze, evaluate and correct in real time the verticality of multiple sclerosis patients during exercise. International Journal of Environmental Research and Public Health 10: 5807-5829.
24. Engebretson J (2002) Culture and complementary therapies. Complementary Therapies in Nursing and Midwifery 8: 177-184.
25. Esmonde L, Long AF (2008) Complementary therapy use by persons with multiple sclerosis: benefits and research priorities. Complement Ther Clin Pract 14: 176-184.
26. Fawcett J, Sidney JS, Hanson MJ, Riley-Lawless K (1994) Use of alternative health therapies by people with multiple sclerosis: an exploratory study. Holist Nurs Pract 8: 36-42.
27. Fowler S, Newton L (2006) Complementary and alternative therapies: the nurse's role. J Neurosci Nurs 38: 261-264.
28. Fryze W, Mirowska-Guzel D, Wiszniewska M, Darda-Ledzion L, Czlonkowska A, et al. (2006) Alternative methods of treatment used by multiple sclerosis patients in Poland. Neurologia i Neurochirurgia Polska 40: 386-390.
29. Galantino ML, Boothroyd C, Lucci S (2003) Complementary and alternative medicine interventions for the orthopedic patient: A review of the literature. Seminars in Integrative Medicine 1: 65-79.
30. Hankin VM (2010) Mindfulness based stress reduction in couples facing multiple sclerosis: Impact on self reported anxiety and uncertainty. Dissertation Abstracts International: Section B: The Sciences and Engineering 70: 6551.
31. Harper E (2009) The effects of pranic breathing on maximal respiratory pressures, fatigue and quality of life of individuals with multiple sclerosis. Dissertation Abstracts International: Section B: The Sciences and Engineering 70: 3470.
32. Hayes M, Chase S (2010) Prescribing Yoga. Primary Care - Clinics in Office Practice 37: 31-47.

33. Hessen C, Romberg A, Gold S, Schulz KH (2006) Physical exercise in multiple sclerosis: Supportive care or a putative disease-modifying treatment. Expert Review of Neurotherapeutics 6: 347-355.
34. Huntley A (2006) A review of the evidence for efficacy of complementary and alternative medicines in MS. International MS Journal 13: 4-12.
35. Jain S, Janssen K, DeCelle S (2004) Alexander technique and Feldenkrais method: A critical overview. Physical Medicine and Rehabilitation Clinics of North America 15: 811-825.
36. Jensen MP, Barber J, Hanley, MA, Raichle KA, Osborne TL,et al. (2007) The effects of self-hypnosis training on pain in persons with multiple sclerosis. FACT 12: 28-29.
37. Jovanov E (2005) On Spectral Analysis of Heart Rate Variability during Very Slow Yogic Breathing. Conf Proc IEEE Eng Med Biol Soc 3: 2467-2470.
38. Kabat-Zinn J (2002) Commentary on Majumdar et al.: Mindfulness meditation for health. Journal of Alternative and Complementary Medicine 8: 731-735.
39. Latimer-Cheung AE, Pilutti LA, Hicks AL, Martin Ginis KA, Fenuta AM, et al. (2013) Effects of exercise training on fitness, mobility, fatigue, and health-related quality of life among adults with multiple sclerosis: A systematic review to inform guideline development. Archives of Physical Medicine and Rehabilitation 94: 1800-1828.
40. Lee JA, Kim JW, Kim DY (2012) Effects of yoga exercise on serum adiponectin and metabolic syndrome factors in obese postmenopausal women. Menopause (New York, NY). pp. 296-301.
41. Lovera J, Kovner B (2012) Cognitive impairment in multiple sclerosis. Current Neurology and Neuroscience Reports 12: 618-627.
42. Mackereth PA, Booth K, Hillier VF, Caress AL (2009) Reflexology and progressive muscle relaxation training for people with multiple sclerosis, A crossover trial. Complement Ther Clin Pract 15: 14-21.
43. Madanmohan, Thombre DP, Balakumar B, Nambinarayanan TK, Thakur S, et al. (1992) Effect of yoga training on reaction time, respiratory endurance and muscle strength. Indian J Physiol Pharmacol 36: 229-233.
44. Maguire BL (1996) Effects of imagery on attitudes and moods in multiple sclerosis patients. Altern Ther Health Med 2: 91-92.
45. Mailhan L, Papeix C (2012) [Non-medicinal treatments of spasticity in multiple sclerosis]. Rev Neurol (Paris) 168 Suppl 3: S57-61.
46. Markil N, Whitehurst M, Jacobs PL, Zoeller RF (2012) Yoga Nidra relaxation increases heart rate variability and is unaffected by a prior bout of Hatha yoga. J Altern Complement Med 18: 953-958.
47. McDonnell MN, Smith AE, Mackintosh SF (2011) Aerobic exercise to improve cognitive function in adults with neurological disorders: a systematic review. Arch Phys Med Rehabil 92: 1044-1052.
48. Meyer HB, Katsman A, Sones AC, Auerbach DE, Ames D, et al. (2012) Yoga as an ancillary treatment for neurological and psychiatric disorders:A review. Journal of Neuropsychiatry and Clinical Neurosciences 24: 152-164.
49. Miller RG (2006) Fatigue and therapeutic exercise. Journal of the Neurological Sciences 242: 37-41.
50. Mishra SK, Singh P, Bunch SJ, Zhang R (2012) The therapeutic value of yoga in neurological disorders. Ann Indian Acad Neurol 15: 247-254.
51. Molina-Rueda F, Pérez de la Cruz S (2009) Multiple sclerosis and relaxation techniques. Esclerosis múltiple y técnicas de relajación 12: 28-34.
52. Monika n, Singh U, Ghildiyal A, Kala S, Srivastava N (2012) Effect of Yoga Nidra on physiological variables in patients of menstrual disturbances of reproductive age group. Indian journal of physiology and pharmacology. pp. 161-167.
53. Muralikrishnan K, Balakrishnan B, Balasubramanian K, Visnegarawla F (2012) Measurement of the effect of Isha Yoga on cardiac autonomic nervous system using short-term heart rate variability. J Ayurveda Integr Med 3: 91-96.
54. Nayak S, Matheis RJ, Schoenberger NE, Shiflett SC (2003) Use of unconventional therapies by individuals with multiple sclerosis. Clinical Rehabilitation 17: 181-191.

55. Oken BS, Flegal K, Zajdel D, Kishiyama SS, Lovera J, et al. (2006) Cognition and fatigue in multiple sclerosis: Potential effects of medications with central nervous system activity. J Rehabil Res Dev 43: 83-90.
56. Ozura A, Jazbec SS (2011) Authors reply. Focus on Alternative and Complementary Therapies 16: 160.
57. Page SA, Verhoef MJ, Stebbins RA, Metz LM, Christopher Levy J (2003) The use of complementary and alternative therapies by people with multiple sclerosis. Chronic Diseases in Canada 24: 75-79.
58. Pal GK, Chandrasekaran A, Hariharan AP, Dutta TK, Pal P, et al. (2012) Body mass index contributes to sympathovagal imbalance in prehypertensives. BMC Cardiovasc Disord 12: 54.
59. Patil NJ, Nagaratna R, Garner C, Raghuram NV, Crisan R (2012) Effect of integrated Yoga on neurogenic bladder dysfunction in patients with multiple sclerosis-A prospective observational case series. Complement Ther Med 20: 424-430.
60. Patra S, Telles S (2010) Heart rate variability during sleep following the practice of cyclic meditation and supine rest. Applied Psychophysiology and Biofeedback 35: 135-140.
61. Pearce CF, Hansen WF (2012) Headache and neurological disease in pregnancy. Clinical Obstetrics and Gynecology 55: 810-828.
62. Penner IK, Schläger R (2006) Fatigue - Better understanding, better therapy? Schweizer Archiv fur Neurologie und Psychiatrie 157: 46-53.
63. Piwko C, Desjardins OB, Bereza BG, Machado M, Jaszewski B, et al. (2007) Pain due to multiple sclerosis: Analysis of the prevalence and economic burden in Canada. Pain Research and Management 12: 259-265.
64. Pozzilli C, Sbardella E, De Giglio L, Tomassini V (2006) Treatment of multiple sclerosis-related fatigue: Pharmacological and non-pharmacological approaches. Neurological Sciences 27: s297-s299.
65. Pritchard M, Elison-Bowers P, Birdsall B (2010) Impact of integrative restoration (iRest) meditation on perceived stress levels in multiple sclerosis and cancer outpatients. Stress and Health: Journal of the International Society for the Investigation of Stress 26: 233-237.
66. Rae-Grant AD, Turner AP, Sloan A, Miller D, Hunziker J, et al. (2011) Self-management in neurological disorders: Systematic review of the literature and potential interventions in multiple sclerosis care. Journal of Rehabilitation Research and Development 48: 1087-1100.
67. Rodgers DM, Beatty WW, MacEachen ME, Khoo KP, Oven MM (1996) Effect of multimodal psycho-spiritual treatment on multiple sclerosis. Altern Ther Health Med 2: 92-93.
68. Rothenberg S, Belok S, Fields JZ (2003) The Maharishi Vedic Medicine Chronic Disorders Program: Introduction and case histories. Alternative and Complementary Therapies 9: 183-190.
69. Ryan M, Johnson MS, Dalmady-Israel C, Kaiser JM (2002) Use of alternative medications in patients with neurologic disorders. Annals of Pharmacotherapy 36: 1540-1545.
70. Salgado BC, Jones M, Ilgun S, McCord G, Loper-Powers M, et al. (2013) Effects of a 4-month Ananda Yoga Program on Physical and Mental Health Outcomes for Persons With Multiple Sclerosis. Int J Yoga Therap 23: 27-38.
71. Schwarz S, Knorr C, Geiger H, Flachenecker P (2008) Complementary and alternative medicine for multiple sclerosis. Mult Scler 14: 1113-1119.
72. Senders A, Wahbeh H, Spain R, Shinto L (2012) Mind-body medicine for multiple sclerosis: a systematic review. Autoimmune Dis 2012: 567324.
73. Shakeel M, Trinidade A, Ah-See KW (2010) Complementary and alternative medicine use by otolaryngology patients: A paradigm for practitioners in all surgical specialties. European Archives of Oto-Rhino-Laryngology 267: 961-971.
74. Shigaki CL, Glass B, Schopp LH (2006) Mindfulness-based stress reduction in medical settings. Journal of Clinical Psychology in Medical Settings 13: 209-216.

75. Shinto L, Yadav V, Morris C, Lapidus JA, Senders A, et al. (2006) Demographic and health-related factors associated with complementary and alternative medicine (CAM) use in multiple sclerosis. Multiple Sclerosis 12: 94-100.
76. Skovgaard L, Nicolajsen PH, Pedersen E, Kant M, Fredrikson S, et al. (2013) People with multiple sclerosis in Denmark who use complementary and alternative medicine-Do subgroups of patients differ? European Journal of Integrative Medicine 5: 365-373.
77. Stevinson C (2011) Can yoga and climbing improve symptoms of multiple sclerosis? Focus on Alternative and Complementary Therapies 16: 159-160.
78. Tavee J (2012) Smoking cessation for the neurologic patient. Neurology: Clinical Practice 2: 112-121.
79. Tavee J, Stone L (2010) Heaing the mind: Mediation and multiple sclerosis. Neurology 75: 1130-1131.
80. Telles S, Raghavendra BR, Naveen KV, Manjunath NK, Kumar S, et al. (2013) Changes in autonomic variables following two meditative states described in yoga texts. J Altern Complement Med 19: 35-42.
81. Thompson AJ (2005) Neurorehabilitation in multiple sclerosis: Foundations, facts and fiction. Current Opinion in Neurology 18: 267-271.
82. Vojdani A, Lambert J, Kellermann G (2011) The role of th17 in neuroimmune disorders: A target for cam therapy. part III. Evidence-based Complementary and Alternative Medicine 2011.
83. Wahbeh H, Elsas SM, Oken BS (2008) Mind-body interventions: applications in neurology. Neurology 70: 2321-2328.
84. Walker ID, Gonzalez EW (2007) Review of intervention studies on depression in persons with multiple sclerosis. Issues Ment Health Nurs 28: 511-531.
85. Wiles CM (2008) Physiotherapy and related activities in multiple sclerosis. Multiple Sclerosis 14: 863-871.
86. Winterholler M, Erbguth F, Neundorfer B (1997) Use of alternative medicine by patients with multiple sclerosis: Users' characterization and patterns of use. Fortschritte der Neurologie, Psychiatrie 65: 555-561.
87. Yadav V, Bourdette D (2006) Complementary and alternative medicine: is there a role in multiple sclerosis? Curr Neurol Neurosci Rep 6: 259-267.
